# Diffusion of neuromodulators for temporal credit assignment

**DOI:** 10.1073/pnas.2608831123

**Published:** 2026-09-14

**Authors:** João Barretto-Bittar, Anna Levina, Emmanouil Giannakakis, Roxana Zeraati

**Affiliations:** ^a^ Department of Computer Sciences, University of Tübingen, Tübingen 72076, Germany; ^b^ Department of Data Analytics and Digitalisation, Maastricht University, Maastricht 6200 MD, Netherlands; ^c^ Department of Computational Neuroscience, Max Planck Institute for Biological Cybernetics, Tübingen 72076; ^d^ Department of Bioengineering, Imperial College London, London SW7 2BP, United Kingdom

**Keywords:** temporal credit assignment, neuromodulators, RNN

## Abstract

Biological learning achieves temporal credit assignment despite sparse and imprecise feedback, often relying on neuromodulatory signals acting over space and time. Here, we introduce a learning mechanism in which error information diffuses locally through the network, similar to volume transmission of neuromodulators. This distributed modulation allows neurons to learn even in the absence of direct feedback, using the local concentration of the diffusing credit signal. Applied to recurrent spiking neural networks with sparse feedback connectivity, diffusive credit signaling improves learning across three benchmark tasks. Using eligibility propagation as a baseline, we show how diffusion-based modulation can provide a plausible mechanism for credit assignment in sparsely connected neural circuits.

Biological learning relies on tightly coordinated local plasticity mechanisms and modulatory systems. In contrast, artificial neural networks (ANNs) are overwhelmingly trained via backpropagation of errors, an exact credit-assignment method that achieves high performance across a wide range of tasks. This success has motivated the hypothesis that biological learning may follow similar principles (1). However, biological constraints (nonexact credit assignment, sparse connectivity and feedback, etc.) make an exact implementation of backpropagation in biological networks unlikely, motivating a search for biologically plausible alternatives.

Eligibility propagation (e-prop) (2) is among the most successful biologically plausible alternatives to backpropagation through time (BPTT). Its performance, however, deteriorates in networks with sparse feedback connectivity, architectures more closely resembling biological networks (3). Recent extensions incorporating neuromodulatory signals have achieved performance improvements by enriching the learning signal with additional structure or cell-type specific communication(4, 5). While the introduced cell-type-specific neuromodulators already depart from synapse-specific weighting, their propagation still follows the connectivity graph precisely. In contrast, neuromodulatory systems predominantly operate through volume transmission, with signals diffusing through the extracellular space and modulating populations of neurons over extended spatial scales (6, 7).

Here, we examine a learning mechanism in which credit signals spread spatially through the network, with credit assignment determined by the local concentration of a diffusible modulatory signal rather than by its point of origin.

## Results

**Fig. 1.**
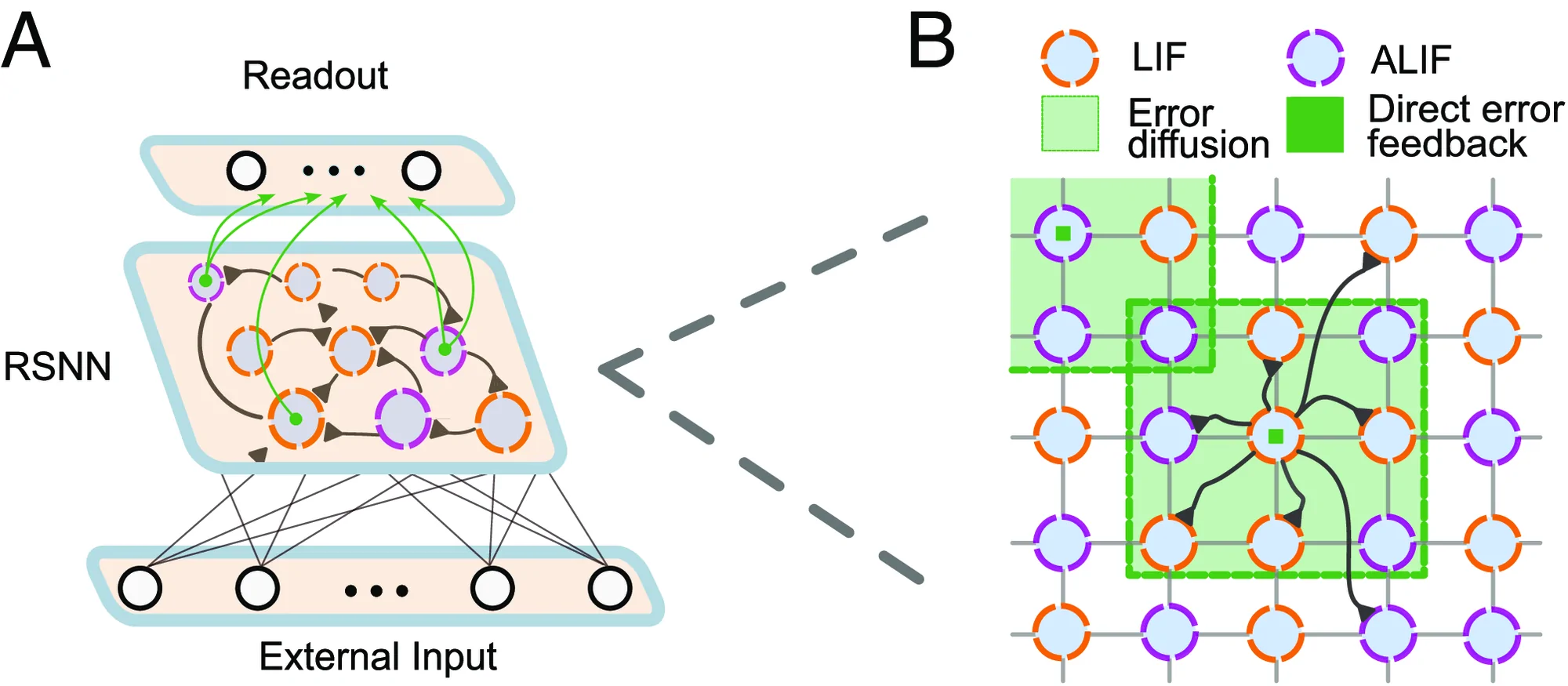
Network architecture and local connectivity pattern. (*A*) A spatially embedded RSNN with local connectivity receives inputs from an external input layer and feeds readout layer to generate the output. The RSNN is sparsely connected to the input and readout layers. (*B*) LIF and ALIF cells are randomly placed in an equally spaced 2D grid and spatially connected. Only a few cells receive direct neuromodulatory feedback. Upon arrival, the neuromodulatory signal diffuses to neighboring neurons.

To assess the impact of diffusing credit signals on learning, we study recurrent spiking neural networks (RSNNs), learning to perform complex temporal tasks. Each RSNN receives task-specific input as spike trains from an external input layer, and its activity is read out by an output layer of leaky nonspiking neurons (Fig. 1*A*).

Our RSNNs consist of two neuron types: leaky integrate-and-fire (LIF) and its variant with firing-rate adaptation (ALIF). The proportion varies across tasks (details in *SI Appendix*). The neurons are randomly embedded on a uniformly spaced 2D grid, where the probability of a connection from neuron *i* to neuron *j* decreases exponentially with the squared distance between them (Fig. 1*B*). The decay rate was set to achieve approximately 10% connectivity. This arrangement promotes a local connectivity pattern, favoring links between nearby neurons. The connectivity to the input and readout layers is sparse, comprising a random 10% subset of all possible connections, without bias toward either neuron type. Additional implementation details and model equations are provided in *SI Appendix*.

During each task, the RSNNs receive feedback credit signals that encode the network’s task-related error, which modulates learning but not neuronal activity. Critically, we assume that neuromodulatory signals do not operate with surgical precision. Rather, once released, they diffuse through intercellular space to influence neighboring cells over several subsequent time steps (Fig. 1*B*). Due to spatial diffusion, the total credit signal available to neuron *j* at time step *t*, $C_{j}^{t,\mathrm{total}}$, is the sum of directly delivered credit signals, $C_{j}^{t,\mathrm{direct}}$, and those arriving via diffusion, $C_{j}^{t,\mathrm{diff}}$. The exact form of the direct credit signal is determined by the learning rule, for example, being proportional to the task loss in e-prop (2). Importantly, the feedback connectivity is also kept sparse (only 10% of the possible connections). As a result, for most neurons, feedback credit signals are available only through diffused credit signals.

To capture biological processes that progressively reduce the availability of released neuromodulators, such as reuptake and enzymatic degradation, we assume that, at each time step, the local concentration of the neuromodulatory signal decays at a fixed rate k∈[0,1] prior to diffusion. Diffusion redistributes the available neuromodulators between the original location and all the neighbors, resulting in the total neuromodulator arriving in the location *i* to be

[1] $$\begin{array}{c} C_{j}^{t,\mathrm{diff}}=\sum_{i\in N_{\left(j\right)}^{M}}D_{\mathrm{ji}}\cdot k\cdot C_{i}^{t-1,\mathrm{total}} \end{array}$$

Here $D_{\mathrm{ji}}$ denotes the fraction of the neuromodulatory signal available at location *i* that is transferred to *j* over one time step, and $N_{\left(j\right)}^{M}$ defines the diffusion neighborhood of neuron *j*. By conservation law, we have that $\sum_{i\in N_{\left(j\right)}^{M}}D_{\mathrm{ji}}=1$. In this work, we adopt the Moore neighborhood, which includes the neuron itself and its eight immediate neighbors. Consequently, a fraction of the available signal remains at neuron *j* at each time step rather than diffusing away. We assume homogeneous diffusion within this neighborhood, such that the available signal is distributed uniformly across all neighbors, i.e., ($D_{\mathrm{ji}}=\frac{1}{9}$).

Our approach can be combined with any learning rule that incorporates feedback signals into its formulation. Here, we adopt eligibility propagation (e-prop) (2), which falls into this category and is one of the state-of-the-art biologically plausible learning rules for RSNNs.

E-prop’s update rule factorizes into two components: a local eligibility trace, $e_{\mathrm{ji}}^{t}$, and a top–down modulation credit signal $C_{j}^{t,\mathrm{total}}$, resulting in $\Delta W_{\mathrm{ji}}=\eta \sum_{t}C_{j}^{t,\mathrm{total}}e_{\mathrm{ji}}^{t},$with *η* being the learning rate. Those two terms are derived so that the update approximates BPTT. Their exact expressions depend on the neuron and network models (for our network, see *SI Appendix*). In short, the eligibility trace acts as a decaying memory of pre- and postsynaptic activity, while the learning signal modulates the magnitude of the weight updates according to the network’s error in the task.

**Fig. 2.**
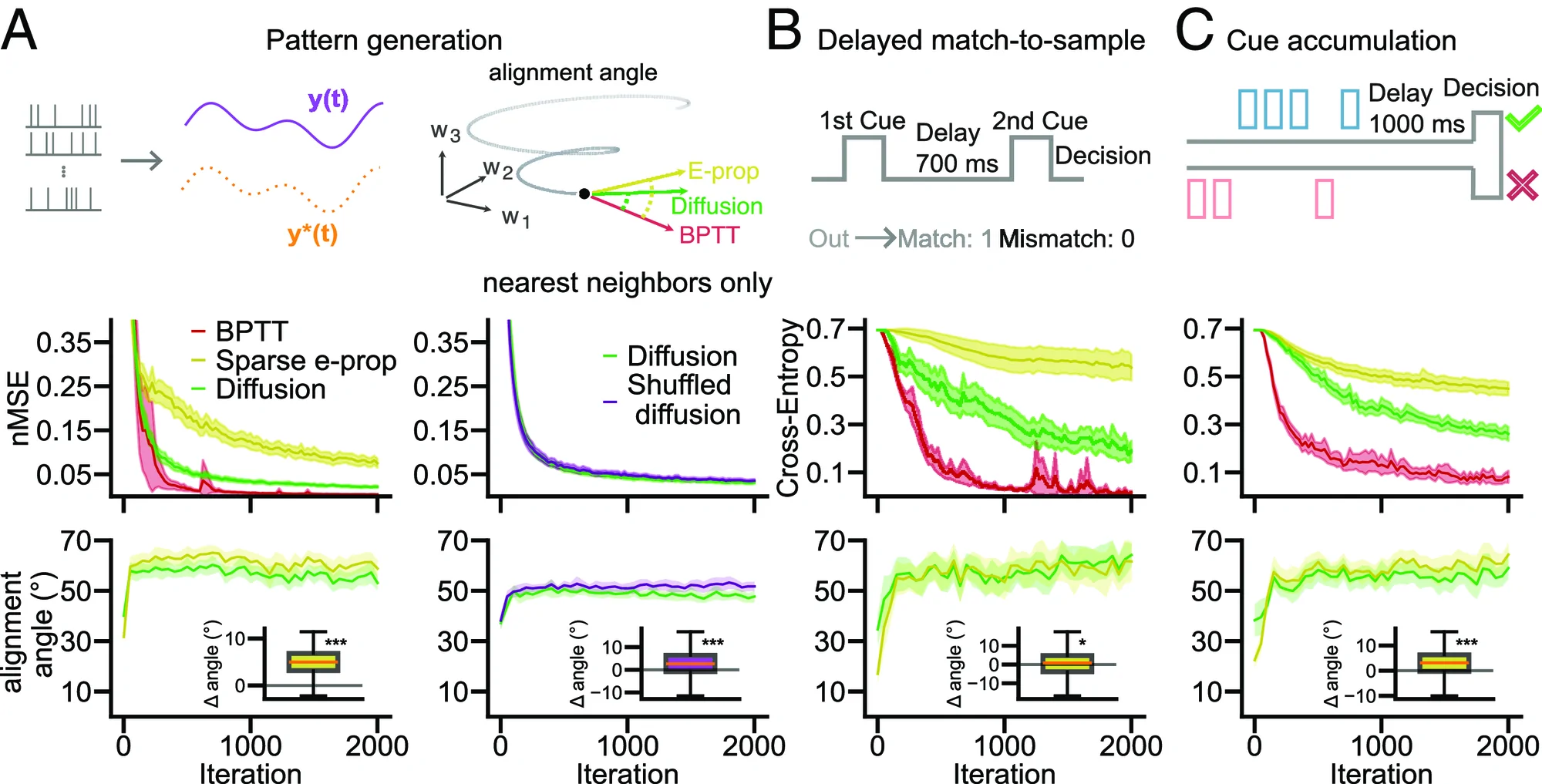
Diffusion of the neuromodulatory signal improves e-prop for RSNN with sparse readout connectivity. Columns (*A*–*C*): (*A*) pattern generation, (*B*) delayed match-to-sample (DMS), (*C*) cue accumulation. Lines/shading show mean ± s.e.m. across 20 runs. *Top*: task schematic (*A*–*C*) and schematic of alignment angle (*A*, *Right*). *Middle*: learning curves for BPTT, e-prop, and e-prop with diffusion (*A*–*C*) or diffusion and shuffled diffusion (*A*, *Right*) [normalized MSE in (*A*)]; shown for decay rate k=0.75. *Bottom*: (*A*–*C*) Gradient alignment angles between BPTT and other algorithms (color-coded as in *Middle* panels, smaller = better alignment). They are computed every 50 iterations at matched network states for recurrent weights; input weight gradients behave similarly. *Insets*: pairwise difference in alignment angle (e-prop − diffusion or shuffled diffusion − diffusion), pooled across runs/iterations (excluding the initialization step). One-sided Wilcoxon signed-rank test: *P<0.05, **P<0.01, and ***P<0.001. Details in *SI Appendix*.

Using e-prop with and without diffusion, we train our networks on three benchmark tasks: pattern generation, delayed match-to-sample (DMS), and cue accumulation (4). In the first task, pattern generation (Fig. 2*A*), the network should learn to reproduce a 1-dimensional target signal composed of a weighted sum of five sinusoids, using realizations of Poisson noise as input. In this task, error feedback is provided at every time step. In contrast, the DMS and cue accumulation tasks provide error signals only at the final time frame, when the network must make a decision based on prior inputs. In the DMS task (Fig. 2*B*), the goal is to compare the values of two binary cues presented with a delay window between them, and then to determine whether the cues were identical (1-1 or 0-0) or different (1-0 or 0-1). Meanwhile, in the cue accumulation task (Fig. 2*C*), a sequence of seven cues is presented, each appearing on either the Left or Right side. Following a delay period with no cue, the network must identify the side with majority of cues.

We find that diffusing error signals improves e-prop performance across all three tasks (Fig. 2) in the sparse-feedback connectivity setup (for dense readout, performances of both variants are comparable). Compared with standard e-prop, our variant consistently yields better learning outcomes, narrowing the performance gap with BPTT.

Moreover, we observe that, over the course of training, diffusion gradients align better with BPTT, producing updates that are directionally more similar to BPTT than those of e-prop without diffusion (Fig. 2). This effect is most pronounced for pattern generation and cue accumulation.

To probe the interaction between local connectivity and proximity-based diffusion, we introduce a shuffled diffusion con-trol in which neurons’ positions in the diffusion grid are randomly permuted at the start of training, disrupting any spatial alignment between the connectivity pattern and the diffusion (*SI Appendix*). We find that learning performance is similar to local diffusion, but local diffusion yields a small yet significant improvement in alignment with BPTT for the pattern generation ($\text{median}\;\Delta \;\text{angle}=2.21^{{}^{\circ}};P=3.00\times 10^{-7}$; one-sided Wilcoxon signed-rank test) and cue accumulation tasks ($\text{median}\;\Delta \;\text{angle}=0.71^{{}^{\circ}};P=4.47\times 10^{-3}$; one-sided Wilcoxon signed-rank test).

To further assess the potential benefits of connectivity matching the diffusion pattern, we repeat this analysis in RSNNs with nearest-neighbor connectivity, in which each neuron connects to exactly its eight closest neighbors on the grid. Learning curves again remain similar across conditions, while the alignment advantage of local diffusion becomes more pronounced for pattern generation (Fig. 2) and cue accumulation ($\text{median}\;\Delta \;\text{angle}=2.27^{{}^{\circ}};\;P=6.39\times 10^{-8}$; one-sided Wilcoxon signed-rank test), but remains nonsignificant for DMS ($\text{median}\;\Delta \;\text{angle}=-1.00^{{}^{\circ}};P=0.09$; one-sided Wilcoxon signed-rank test). These results indicate that in the examined tasks, spatial alignment between connectivity and diffusion is not generally necessary, but can become beneficial under certain conditions.

## Discussion

Temporal credit assignment with sparse feedback is challenging, and even state-of-the-art biologically plausible rules, such as e-prop, struggle in this setting. Random e-prop (2), using random feedback weights, performs well in sparse networks, yet assumes each neuron receives a dedicated error signal. E-prop augmented with cell-type-specific local neuromodulatory signals also improves performance under sparse feedback (4), but still relies on direct communication of errors between connected neurons. Here, we show that a less precise form of neuromodulatory communication, based on chemical diffusion, can benefit local learning in circuits that lack specialized cell-type-specific signaling. This enhancement emerges specifically under sparse feedback connectivity and would largely disappear with densely connected feedback. The diffusion mechanism can also be extended to incorporate diverse neuromodulators, reconciling the benefits of specialized signaling with the broader reach of volume transmission.

We found that diffusion improves gradient alignment potentially through a feedback-alignment-like effect: diffusion training dynamics progressively bias the effective feedback pathway toward directions correlated with the true gradient, despite lacking sign- or weight-specific information. This resembles how random feedback weights align in standard feedback alignment (8). This mechanism appears to work even with shuffled diffusion. However, the alignment with the true gradient improves further with local diffusion in certain tasks, which may require more temporally precise recurrent dynamics. Future work on complex tasks where network topology and long-term temporal credit assignment are expected to play a greater role is needed to mechanistically account for how diffusion improves gradient alignment and interacts with connectivity structure and task demands.

While it is well established that the brain relies on both synaptic and volume transmission for neuromodulation (9), the latter has received less attention in ANNs. It has been suggested that such a mechanism can help mitigate catastrophic forgetting (10) and increase dynamical flexibility of the network by selectively modulating subsets of neurons (11). Recent studies have also demonstrated that volume transmission of modulatory signals can enable gating properties and implement context factorization in RNNs (12, 13).

Our results suggest that biochemical processes known to operate in biological circuits, such as the diffusion of modulatory substances, may play a functional role in enabling learning under realistic connectivity constraints. Our findings motivate further investigation into the interaction between neuromodulator dynamics and learning in biological systems, particularly for spatially embedded artificial networks.

## Materials and Methods

We trained sparsely connected RSNNs using e-prop augmented with spatiotemporal diffusion of credit signals (details in *SI Appendix*).

## Supplementary Material

Appendix 01 (PDF)

## Data Availability

Scripts have been deposited in GitHub (LevinaLab/neuromodRNNs) (14).
