# Supplementary material for: Diffusion of neuromodulators for temporal credit assignment
Source: Proc Natl Acad Sci U S A. 2026 Sep 14;123(38):e2608831123. doi: 10.1073/pnas.2608831123 (PMC13598169; doi:10.1073/pnas.2608831123)
Supplement: Supplementary file 1 [file pnas.2608831123.sapp.pdf]

Supplementary Information: Diffusion of Neuromodulators for Temporal Credit Assignment

Joao Barretto-Bittar, Anna Levina, Emmanouil Giannakakis, Roxana Zeraati
(Dated: August 17, 2026)

I. Network Model

All simulations reported in this work are based on recurrent spiking neural networks (RSNNs) that receive spike-based inputs from an external input layer. An output layer reads out the activity of the RSNN and produces the network output.

A. Spiking Neurons

In line with [1], we adopt discrete-time recurrent spiking neural networks (RSNNs), consisting of a combination of leaky integrate-and-fire (LIF) and adaptive threshold LIF (ALIF) neurons [2]. The overall network size and the proportion of LIF and ALIF neurons vary across tasks, with full architectural details provided in Section IX.

The dynamics of the LIF and ALIF neurons in the recurrent layer are governed by the following equation for the membrane potential v_j^t

$$v_j^t = \alpha v_j^{t-1} + (1-\alpha)(\sum_{i \neq j} W_{ji}^{\text{rec}} z_i^{t-1} + \sum_i W_{ji}^{\text{in}} x_i^t) - z_j^{t-1} v_{\text{th}}, \tag{1}$$

with $\alpha = e^{\Delta t/\tau_m}$. Here Δt is the simulation time step, fixed to 1 ms in all experiments, and $\tau_m \in \{20\,\text{ms}, 30\,\text{ms}\}$ is the membrane time constant.

We assume a transmission delay of 1ms for recurrent connections, such that recurrent spikes influence the postsynaptic membrane potential at the next time step, as reflected by the term z_i^{t-1} in Eq. (1). In contrast, external inputs are applied without delay and therefore enter the dynamics at the current time step via x_i^t.

W_{ji}^{rec} and W_{ji}^{in} denote the synaptic weights from presynaptic neuron i to postsynaptic neuron j, where neuron i belongs to the recurrent layer or the external input layer, respectively. The variable z_j^t indicates whether neuron j emits a spike at time t and is defined as

$$z_j^t = H\left(v_j^t - A_j^t\right), \tag{2}$$

where $H(\cdot)$ is the Heaviside step function. Therefore, a spike z_j^t is emitted whenever the membrane potential v_j^t exceeds the neuron's instantaneous firing threshold A_j^t, whose dynamics is given by

$$A_j^t = v_{\text{th}} + \beta_j a_j^t, \tag{3}$$

where v_{th} is a baseline threshold and the variable β_j controls the strength of spike-triggered adaptation by scaling the contribution of an adaptation variable a_j^t. The adaptation dynamics follows

$$a_j^t = \rho a_j^{t-1} + (1-\rho) z_j^{t-1}, \tag{4}$$

with $\rho = e^{\Delta t/\tau_{\text{adapt}}}$ denoting the decay factor of the adaptation variable. The adaptation time constant τ_{adapt} is chosen to match the temporal scale of the working memory demands of the task; for example, in the cue accumulation task, we set $\tau_{\text{adapt}} = 2000\,\text{ms}$.

For LIF neurons, the parameter β_j in 3 is set to zero, effectively keeping the firing threshold fixed at its baseline value v_{th}. Additionally, as in [1, 3], we implement a simple model of the refractory period where after neuron j emits a spike, all state variables are allowed to evolve freely, except for the spike output z_j^t, which is clamped to zero for a short period of 2–5 ms, depending on the task. A detailed description of the parameter choices across tasks is provided in Section IX.

B. Network Output and Task Loss Function

The network output y_k^t is generated by leaky non-spiking readout neurons, with dynamics described by

$$y_k^t = \kappa y_k^{t-1} + (1-\kappa)\left(\sum_j W_{kj}^{\text{out}} z_j^t\right), \tag{5}$$

where W_{kj}^{out} denotes the synaptic weight from recurrent neuron j to readout neuron k. The parameter $\kappa = e^{\Delta_t/\tau_{\text{out}}}$ is the leak factor of the output neurons, with membrane time constant τ_{out} set equal to the recurrent membrane time constant τ_m in all simulations.

Network performance is evaluated using a task-dependent loss function, defined in Eq. 6:

$$E^{\text{task}}(y, y^*) = \begin{cases} \frac{1}{2}\sum_{k,t}\left(y_k^{*,t} - y_k^t\right)^2, & \text{for regression tasks,} \\ -\sum_{k,t}\pi_k^{*,t}\log \pi_k^t, & \text{for classification tasks.} \end{cases} \tag{6}$$

Here, $y_k^{*,t}$ denotes the target value for regression tasks, while $\pi_k^{*,t}$ is the one-hot encoded target label for classification tasks. The predicted class probabilities π_k^t are obtained by applying a softmax transformation to the readout activities, Eq. 7.

$$\pi_k^t = \frac{\exp(y_k^t)}{\sum_{k'}\exp(y_{k'}^t)}. \tag{7}$$

C. Connectivity and Spatial Embedding

All RSNNs employ fixed, random sparse connectivity for the external input and readout weights, and a fixed distance-dependent local connectivity pattern for the recurrent weights. This is implemented by defining a binary connectivity matrix A_{ji}^{layer} for each layer, where $A_{ji}^{\text{layer}} = 1$ indicates the presence of a synaptic connection from presynaptic neuron i to postsynaptic neuron j, and $A_{ji}^{\text{layer}} = 0$ denotes the absence of a connection. Once initialized, each connectivity matrix A_{ji}^{layer} is kept fixed throughout training, and no structural plasticity or rewiring is allowed. Corresponding weights W_{ji}^{layer} with $A_{ji}^{\text{layer}} = 0$ are set to zero and excluded from optimization.

The connectivity matrices A_{ji}^{in} and A_{ji}^{out} for the external input and readout layers are initialized randomly, with 10% of entries set to one. For the recurrent layer, the probability of a synaptic connection from neuron i to neuron j is defined as a function of the spatial distance d_{ji} between the two neurons, with connection probability decreasing exponentially with the squared distance

$$P(A_{ji}^{rec} = 1) = \frac{1}{1 + e^{d_{ji}^2/(4\sigma)}}, \tag{8}$$

where the parameter σ controls the spatial decay of the connection probability. In all simulations, σ is fixed to 0.012, resulting in an average recurrent connectivity of approximately 10%.

To compute inter-neuronal distances, neurons in the recurrent layer are embedded in a two-dimensional grid with h rows and w columns, uniformly distributed over the unit square $\mathbb{I}^2 = [0,1]\times[0,1]$. Each neuron i, irrespective of whether it is an LIF or ALIF unit, is assigned a unique grid position (x_i, y_i) without replacement, ensuring that each spatial location is occupied by at most one neuron. The distance d_{ji} between neurons i and j is defined as the Euclidean distance under periodic boundary conditions:

$$d_{ji} = \sqrt{\min(|x_i - x_j|, 1 - |x_i - x_j|)^2 + \min(|y_i - y_j|, 1 - |y_i - y_j|)^2}. \tag{9}$$

II. E-prop

Bellec et al. [1] introduced e-prop as an approximation to backpropagation through time (BPTT) for recurrent spiking neural networks, motivated by the goal of deriving a more biologically plausible learning rule. An equivalent

learning principle for rate-based recurrent neural networks, termed random feedback local online learning (RFLO), was independently proposed by Murray [4]. The central idea underlying e-prop and RFLO, is to truncate the BPTT factorization, thereby enabling online and local weight updates. In this formulation, synaptic updates depend on three factors: the activity of the pre- and postsynaptic neurons, and a neuron-specific feedback signal conveying information about the global network error. The complete derivation for e-prop updates is described in [1]. Here, we briefly summarize its weight-update rules.

A. Eligibility trace

In e-prop, the eligibility trace associated with a synapse connecting presynaptic neuron i to postsynaptic neuron j is defined as the derivative of the postsynaptic neuron's observable state, here, the spike output z_j^t, with respect to the synaptic weight W_{ji}. For adaptive leaky integrate-and-fire (ALIF) neurons, this definition leads to an eligibility trace that depends on multiple internal state variables. Specifically, the eligibility trace e_{ji}^t can be decomposed into two components as

$$e_{ji}^t = \psi_j^t(e_{ji,v}^t - \beta_j e_{ji,a}^t), \tag{10}$$

where $e_{ji,v}^t$ corresponds to the contribution of the membrane potential variable v_j, given by

$$e_{ji,v}^t = \mathcal{F}_\alpha(z_i^{t-1}), \tag{11}$$

and $e_{ji,a}^t$, corresponds to the contribution of the adaptation variable a_j, given by

$$e_{ji,a}^t = \psi_j^{t-1}(1-\rho)e_{ji,v}^{t-1} + (\rho - (1-\rho)\beta_j\psi_j^{t-1})e_{ji,a}^{t-1}. \tag{12}$$

Here $\mathcal{F}_\kappa$ is a low-pass filter with exponential smoothing $\mathcal{F}_\kappa(x^t) = \kappa\mathcal{F}_\kappa(x^{t-1}) + (1-\kappa)x^t$ and ψ_j^t is a surrogate gradient for $\frac{dz_j}{dv_j}$, defined as

$$\psi_j^t = \begin{cases} 0 & \text{during refractory period,} \\ \frac{\gamma}{v_{\text{th}}}\max(0, 1 - \frac{v_j^t - A_j^t}{v_{\text{th}}}) & \text{otherwise} \end{cases} \tag{13}$$

with γ, a dampening factor, set to 0.3. The use of a surrogate gradient is necessary since the gradient of Heaviside step function is zero everywhere, except at zero, where it is not defined.

We notice that the same equations can be used by LIF neurons, by just setting $\beta_j = 0$. The equations presented below describe the eligibility traces for recurrent synapses. The eligibility traces for external input synapses can be obtained analogously by replacing the presynaptic spike variable z_i^{t-1} with the external input spike x_i^t.

B. Credit Signal

E-prop defines the credit signal C_j^t (referred to as the *learning signal* in [1]) as the truncated derivative of the loss function E with respect to the spike output z_j^t. The truncation ensures that the credit signal can be computed online and locally.

For regression tasks, the feedback credit signal $C_j^{t,\text{direct}}$ is computed as

$$C_j^{t,\text{direct}} = \sum_k W_{kj}^{\text{out}} \left(y_k^{*,t} - y_k^t\right), \tag{14}$$

where W_{kj}^{out} denotes the readout weight connecting neuron j to output unit k. We note that for neurons that are connected to the readout, this direct credit signal is absent (no feedback).

For classification tasks, the same expression applies by substituting the regression targets and outputs $y_k^{*,t}$ and y_k^t with the corresponding class labels $\pi_k^{*,t}$ and predicted probabilities π_k^t, respectively.

We note that our simulations assume symmetric feedback weights W_{kj}^{out}, as we focus on intra-network credit propagation. More biologically plausible alternatives, such as random feedback alignment [5], can be adopted for addressing this problem".

C. Update

The updates for the recurrent and external input weights are calculated as a sum over time of the product of two key components: the credit signal C_j^t (called the learning signal in the original paper) and the eligibility trace e_{ji}^t,

$$\Delta W_{ji}^{\mathrm{rec/in}} = \eta \sum_t C_j^t \mathcal{F}_\kappa(e_{ji}^t), \tag{15}$$

where η is the learning rate and $\mathcal{F}_\kappa$ is the low-pass filter. As shown in the supplemental materials of [1], the low-pass filtering of the eligibility trace is necessary for the online implementation of e-prop, due to the use of leaky readout neurons. We note that, to simplify notation, Eq. 15 shows the update for the vanilla gradient descent optimizer, in which the learning rate is fixed. In our simulations, the updates are implemented with Adam [6].

In the setup with diffusion, we use $C_j^t = C_j^{t,direct} + C_j^{t,diff}$ as explained in Equation 1 in the main text.

The updates for the readout layers are computed as

$$\Delta W_{kj}^{\mathrm{out}} = \eta \sum_t \left(y_k^{*,t} - y_k^t\right) \mathcal{F}_\kappa(z_j^t). \tag{16}$$

III. Firing rate regularization

We include a firing-rate regularization term, following the approach described in the supplementary material of [1]. This regularization penalizes deviations of the average firing rate of recurrent neurons from a target value, thereby encouraging stable and biologically plausible activity levels. The regularization loss is added directly to the task loss. The firing-rate regularization loss is defined as

$$E^{\mathrm{reg}} = \frac{c_{\mathrm{reg}}}{2} \sum_j \left(f_j^{\mathrm{av}} - f^{\mathrm{target}}\right)^2, \tag{17}$$

where f_j^{av} denotes the average firing rate of neuron j computed across all trials in a batch, and f^{target} is the desired target firing rate. In all simulations, f^{target} is fixed to 10 Hz. The adopted regularization coefficient c_{reg} differs across tasks, with specific values reported in Section IX.

The average firing rate f_j^{av} is computed as

$$f_j^{\mathrm{av}} = \frac{1}{BT\Delta t} \sum_{b=1}^{B} \sum_{t=1}^{T} z_j^{t,b}, \tag{18}$$

where B is the batch size, T is the number of simulation time steps per trial, Δt is the simulation time step, and $z_j^{t,b}$ denotes the spike output of neuron j at time step t in trial b.

Under the e-prop framework, this formulation leads to the update

$$\Delta W_{ji}^{\mathrm{rec/in,reg}} = \eta\, c_{\mathrm{reg}} \sum_t \frac{1}{BT\Delta t} \left(f_j^{\mathrm{target}} - f_j^{\mathrm{av}}\right) e_{ji}^t. \tag{19}$$

This contribution is then added to the task-dependent update. Importantly, since the firing-rate regularization term does not rely on a feedback signal from the output layer, its implementation is identical with or without diffusion. Moreover, because this term depends only on local neuronal activity, the resulting update is equivalent to the one obtained via BPTT for the corresponding component of the loss function.

IV. Tasks and Training

A. Tasks

We evaluated the proposed learning rule by comparing its performance with BPTT and e-prop across the three benchmark tasks considered in [3], with slight modifications. The three tasks are detailed below.

1. Pattern Generation

In the pattern generation task, the network is trained to reproduce a one-dimensional continuous target signal from spike-based input. The target signal is constructed as a weighted sum of five sinusoidal components. The network has to learn to generate the same target signal in response to eight distinct but fixed realizations of Poisson noise input. Using multiple fixed input realizations increases task difficulty.

For this task, we use an RSNN composed of 400 LIF neurons with a membrane time constant $\tau_m = 30\,\text{ms}$. The external input layer consists of 100 neurons generating independent Poisson spike trains with a firing rate of 50 Hz. The readout layer comprises a single leaky non-spiking unit with $\tau_{\text{out}} = 20\,\text{ms}$. The firing threshold is set to $v_{\text{th}} = 0.03$.

Each trial lasts 2000 ms. The target signal is generated as a weighted sum of five sinusoids with fixed frequencies of 0.5 Hz, 1 Hz, 2 Hz, 3 Hz, and 4 Hz. For each run, the weights of the individual sinusoidal components are drawn randomly and normalized to sum to one. The resulting signal is then centered to have a zero mean.

2. Delayed Match-to-Sample

The delayed match-to-sample task requires the network to classify whether two sequentially presented cues, separated by a temporal delay, belong to the same category. Each cue can take one of two values, 0 or 1. The network is required to output 1 if both cues are identical (i.e., 1–1 or 0–0), and 0 otherwise (i.e., 1–0 or 0–1).

We employ an RSNN composed of 50 LIF neurons and 50 ALIF neurons, with membrane and adaptation time constants $\tau_m = 20\,\text{ms}$ and $\tau_{\text{adapt}} = 1400\,\text{ms}$, respectively. The adaptation strength parameter is set to $\beta = 1.8$, and the firing threshold is fixed to $v_{th} = 0.03$. The readout layer consists of two output neurons, one representing the probability of returning 1 (matched cues) and the other 0 (unmatched cues). Although binary classification can in principle be implemented using a single readout neuron, we retain two output units for consistency with [1, 3].

Each trial begins with a 50 ms fixation period, followed by the presentation of the first cue. Each cue is presented for 150 ms, with a 700 ms delay separating the two cues. Immediately after the offset of the second cue, a 50 ms decision period is initiated. Cue values are drawn independently and randomly for each trial, with probability $p = 0.5$ of being 1.

External input is provided by four distinct neuronal populations, each consisting of 20 neurons and generating Poisson spike trains. Two populations encode the cue values, firing at 40 Hz when the corresponding cue equals 1 and remaining silent otherwise. A third population encodes the decision period, firing at 40 Hz exclusively during this interval. Finally, a fourth population provides background activity, firing at 10 Hz throughout the entire trial to prevent the network from becoming quiescent during the delay period.

3. Cue Accumulation

In the cue accumulation task, a sequence of seven cues is presented sequentially, each separated by a brief temporal gap. Each cue appears on either the left or the right side. After the presentation of the final cue, a longer delay period follows, after which the agent must report on which side the majority of cues appeared, with 1 indicating the right side and 0 the left side.

We employ the same RSNN architecture as in the delayed match-to-sample task, consisting of 50 LIF and 50 ALIF neurons. The only modification concerns the adaptation time constant, which is set to $\tau_{adapt} = 2000\,\text{ms}$ to accommodate the longer temporal integration demands of this task.

Each cue is presented for 100 ms, followed by a 50 ms inter-cue interval. The side on which each cue appears is drawn independently and randomly for each cue and trial, with equal probability for both sides. After the final cue, a fixed delay period of 1000 ms is imposed, followed by a decision period lasting 150 ms.

External input is provided by four neuronal populations, each consisting of 10 neurons and generating Poisson spike trains. One population is activated whenever a cue appears on the left side, and another when a cue appears on the right side, both firing at a rate of 40 Hz. In addition, a background noise population and a decision-time population are included, identical to those used in the delayed match-to-sample task.

B. Training and Weight Updates

For all tasks, the weights of each layer were initialized using Kaiming normal initialization [7] and subsequently scaled by a layer-specific gain factor.

For the delayed match-to-sample and cue accumulation tasks, learning is performed using a cross-entropy loss, with target labels provided exclusively during the decision period. Consequently, credit signals are available only during this time window. Training is performed using batches of 64 trials, with gradients averaged within each batch before applying weight updates.

For the pattern generation task, learning is based on a mean-squared error loss. Each batch consists of 8 trials, where each trial receives a distinct realization of Poisson input noise; however, these input realizations are fixed across batches. Across all tasks, independently of the specific learning rule, parameter updates are performed using the Adam optimizer [6].

For the delayed match-to-sample and cue accumulation tasks, performance was evaluated on a test set comprising 512 trials generated independently of the training data.

For the pattern generation task, the Poisson input realizations were fixed throughout training and evaluation. Consequently, performance was assessed using the same input realizations as during training.

V. Gradient Alignment Angle

To measure the similarity in directionality between a rule's proposed update and the BPTT update, we adopt the alignment angle

$$\theta \;=\; \arccos\left(\frac{\mathbf{g}_{\text{rule}} \cdot \mathbf{g}_{\text{BPTT}}}{\|\mathbf{g}_{\text{rule}}\|\,\|\mathbf{g}_{\text{BPTT}}\|}\right), \tag{20}$$

where $\mathbf{g}_{\text{rule}}, \mathbf{g}_{\text{BPTT}} \in \mathbb{R}^n$ are the flattened gradient vectors of the rule-proposed and BPTT updates, respectively, $\cdot$ denotes the dot product, $\|\cdot\|$ denotes the ℓ_2 norm, and $\theta \in [0^\circ, 180^\circ]$ is the angle between the two update directions. Any 2D parameter matrices are flattened into 1D vectors prior to computing θ.

To compare alignment differences between e-prop with and without diffusion, RSNNs are trained with diffusion, and every 50 iterations, gradients for both e-prop variants are computed at the pre-update network state and compared against the BPTT gradient. This yields pairwise differences at matching network states and task examples. Because the diffusive term enters the credit signals additively, any difference in alignment angle can be attributed solely to its effect, since the direct credit signal is identical across both variants.

For the comparison between spatially aligned and shuffled diffusion, each variant is trained under its own dynamics. This is necessary for a fairer comparison, as the diffusion term aligns to the BPTT gradient during the initial training iterations. In this case, pairwise differences are obtained at matched iterations and initialisation random seeds. However, since each variant evolves under its own dynamics, identical network states are not guaranteed, even when learning curves are comparable.

In the inset box plots of Fig.2, whiskers extend to the most extreme data point within 1.5× the interquartile range (IQR) from the box, and outliers beyond this range are excluded from the plots but included in statistical testing. Statistical significance is assessed using a one-sided Wilcoxon signed-rank test. Alignment values at network initialisation are included in the alignment curves but excluded from the box plots and statistical tests.

In all alignment angle analyses, c_{reg} is set to 0. This is because all learning rules compute the gradient with respect to the firing rate regularisation loss E^{reg} exactly, and including it could mask differences in the task-dependent update component, which varies across rules.

VI. Shuffle Diffusion

In the shuffle diffusion control, the RSNNs are embedded in a 2-D grid and assigned the same connectivity pattern as in the spatial aligned diffusion condition. However, the diffusion operates on a separate grid in which each neuron occupies a randomly permuted position, that is, neuron j is placed at a different grid location than it occupies in the network grid. This permutation is drawn once per run and held fixed throughout all training iterations.

As a result, an error signal injected at a given location in the diffusion grid spreads to the same set of locations at every time step, yet those locations no longer correspond to the neuron's spatial neighbors in the network grid. In this way, any spatial alignment between the connectivity pattern and the diffusion spread is disrupted.

This manipulation therefore preserves both the network topology and the structure of the diffusion operator, ensuring that any observed differences can be attributed specifically to the spatial alignment between the diffusion grid and the network grid, and hence with connectivity, rather than to any other architectural or dynamical differences between conditions. We also note that direct feedback signals are not affected by the permutation.

VII. Nearest-Neighbor Connectivity

As an alternative to the distance-dependent probabilistic connectivity described above, we also consider a deterministic nearest-neighbor connectivity pattern for the recurrent layer. In this scheme, each neuron i is connected to and from exactly the 8 neurons occupying the adjacent grid positions in the Moore neighborhood. Boundary conditions are handled periodically, consistent with Equation 9, so that neurons on the edge of the grid are connected to neurons on the opposite edge.

VIII. Code Implementation

All simulations are implemented in Python 3.12.3, using JAX library [8] as the primary framework. The implementation will be released in a public repository upon publication.

IX. Simulation Parameters

Unless otherwise stated, all simulations used a simulation time step of $\Delta t = 1\,\text{ms}$, diffusion constant $k = 0.75$, membrane threshold $v_{\text{th}} = 0.03$, spatial decay parameter for connection probability $\sigma = 0.012$, and surrogate gradient dampening factor $\gamma = 0.3$.

A. Pattern Generation Task

a. Architecture. The recurrent network consisted of 400 LIF neurons and no ALIF neurons, arranged on a 20×20 grid. A single readout neuron was used.

b. Neuron model. Membrane time constants were $\tau_m = 30\,\text{ms}$ and $\tau_{\text{out}} = 30\,\text{ms}$. The refractory period was 2 ms.

c. Training. Training was performed with batch size 8 using mean squared error (MSE) loss. The learning rate was $\eta = 0.010$. The regularization coefficient was $c_{\text{reg}} = 0.010$. Weight initialization gains (input, recurrent, output) were (1.0, 1.0, 1.0). The test set size was 8.

d. Temporal structure. Each trial lasted 2000 ms. A fixed set of 8 input realizations was used throughout training. Target signals consisted of weighted sums of sinusoids with frequencies 0.5, 1, 2, 3, and 4 Hz, normalized such that the weights summed to 1 and the signal had zero mean. Weights were randomly drawn for each different seed run.

e. Input encoding. The input comprised one population of 100 Poisson neurons firing at 50 Hz.

B. Delayed Match-to-Sample

a. Architecture. The recurrent network consisted of 50 LIF and 50 ALIF neurons arranged on a 10×10 grid, with two readout neurons.

b. Neuron model. Membrane time constants were $\tau_m = 20\,\text{ms}$ and $\tau_{\text{out}} = 20\,\text{ms}$. ALIF neurons had adaptation time constant $\tau_{\text{adapt}} = 1400\,\text{ms}$ and adaptation strength $\beta = 1.8$. The refractory period was 5 ms.

c. Training. Training used batch size 64 and cross-entropy loss. The learning rate was $\eta = 0.005$. Two values of the regularization coefficient were evaluated: $c_{\text{reg}} \in \{0.010, 0.100\}$. The higher value (0.100) yielded better performance for BPTT, whereas the lower value (0.010) performed better for e-prop (with and without diffusion). Results in the main text report the best-performing value for each learning rule. Weight initialization gains (input, recurrent, output) were (0.5, 0.1, 0.5). The test set size was 512.

d. Temporal structure. Each trial consisted of a 50 ms fixation period, followed by a 150 ms cue presentation, a 700 ms delay period, a second 150 ms cue presentation and a 50 ms decision period. Cue identity was sampled with probability $p = 0.5$.

e. Input encoding. Four input populations were used, each containing 20 neurons. Cue populations fired at 40 Hz when the respective cue was active. The decision population fired at 40 Hz, and the background population at 10 Hz.

C. Cue Accumulation Task

a. Architecture. The recurrent network consisted of 50 LIF and 50 ALIF neurons arranged on a 10×10 grid, with two readout neurons.

b. Neuron model. Membrane time constants were $\tau_m = 20\,\text{ms}$ and $\tau_{\text{out}} = 20\,\text{ms}$. ALIF neurons had $\tau_{\text{adapt}} = 2000\,\text{ms}$ and $\beta = 1.8$. The refractory period was 5 ms.

c. Training. Training used batch size 64 and cross-entropy loss. The learning rate was $\eta = 0.005$, and the regularization coefficient was $c_{\text{reg}} = 0.005$. Weight initialization gains (input, recurrent, output) were (1.0, 1.0, 1.0). The test set size was 512.

d. Temporal structure. Each trial consisted of 7 sequential cues, each lasting 100 ms and separated by 50 ms inter-cue intervals. After the final cue, a 1000 ms delay preceded a 150 ms decision period. Cue side was sampled with equal probability $p = 0.5$ for each side.

e. Input encoding. Four input populations were used, each containing 10 neurons. Left and right cue populations fired at 40 Hz. The decision population fired at 40 Hz, and the background population at 10 Hz.

X. Hyperparameter Exploration

To verify that the observed learning acceleration of e-prop with diffusion over the baseline model is not an artifact of hyperparameter selection, we performed a grid search over the learning rate η and firing rate regularization coefficient c_{reg}. For each combination, we ran 5 simulations with different random seeds. Across all tasks, when comparing each method at its best hyperparameter setting, diffusion accelerated learning in the three tasks. Moreover, no tested combination allowed the model without diffusion to clearly outperform the one with diffusion. None of the random number generator seeds used during the grid search were reused in other analyses. The grids used for each task are reported below.

a. Pattern Generation $(\eta, c_{\text{reg}}) \in \{0.005,\ 0.010,\ 0.050,\ 0.100\} \times \{0.0001, 0.0010,\ 0.01000,\ 0.1000\}$

b. Delayed Match-to-Sample $(\eta, c_{\text{reg}}) \in \{0.001,\ 0.005,\ 0.010,\ 0.050\} \times \{0.0001, 0.0010, 0.0100,\ 0.1000\}$

c. Cue Accumulation $(\eta, c_{\text{reg}}) \in \{0.001, 0.005,\ 0.010,\ 0.050\} \times \{0.00001, 0.00010, 0.00500,\ 0.05000\}$

[1] G. Bellec, F. Scherr, A. Subramoney, E. Hajek, D. Salaj, R. Legenstein, and W. Maass, A solution to the learning dilemma for recurrent networks of spiking neurons, Nature communications **11**, 3625 (2020).

[2] C. Teeter, R. Iyer, V. Menon, N. Gouwens, D. Feng, J. Berg, A. Szafer, N. Cain, H. Zeng, M. Hawrylycz, *et al.*, Generalized leaky integrate-and-fire models classify multiple neuron types, Nature communications **9**, 709 (2018).

[3] Y. H. Liu, S. Smith, S. Mihalas, E. Shea-Brown, and U. Sümbül, Cell-type–specific neuromodulation guides synaptic credit assignment in a spiking neural network, Proceedings of the National Academy of Sciences **118**, e2111821118 (2021).

[4] J. M. Murray, Local online learning in recurrent networks with random feedback, Elife **8**, e43299 (2019).

[5] T. P. Lillicrap, D. Cownden, D. B. Tweed, and C. J. Akerman, Random synaptic feedback weights support error backpropagation for deep learning, Nature communications **7**, 13276 (2016).

[6] D. P. Kingma and J. Ba, Adam: A method for stochastic optimization, arXiv e-prints , arXiv (2014).

[7] K. He, X. Zhang, S. Ren, and J. Sun, Delving deep into rectifiers: Surpassing human-level performance on imagenet classification, in *Proceedings of the IEEE international conference on computer vision* (2015) pp. 1026–1034.

[8] J. Bradbury, R. Frostig, P. Hawkins, M. J. Johnson, C. Leary, D. Maclaurin, G. Necula, A. Paszke, J. VanderPlas, S. Wanderman-Milne, and Q. Zhang, JAX: composable transformations of Python+NumPy programs (2018).
